# Value of Bile Acids in Diagnosing Hepatitis C Virus-Induced Liver Cirrhosis and Hepatocellular Carcinoma

**DOI:** 10.3389/bjbs.2021.10191

**Published:** 2022-01-10

**Authors:** Ashraf Khalil, Azza ElSheashaey, Eman Abdelsameea, Manar Obada, Mohamed Bayomy F.F., Hala El-Said

**Affiliations:** ^1^ Department of Biochemistry and Molecular Diagnostics, National Liver Institute, Menoufia University, Shibin el Kom, Egypt; ^2^ Department of Zoology, Faculty of Science, Menoufia University, Shibin el Kom, Egypt; ^3^ Department of Hepatology and Gastroenterology, National Liver Institute, Menoufia University, Shibin el Kom, Egypt

**Keywords:** cirrhosis, hepatocellular carcinoma, liquid chromatography-mass spectrometry, metabolic profiling, bile acid

## Abstract

**Background:** Metabonomic studies have related bile acids to hepatic impairment, but their role in predicting hepatocellular carcinoma still unclear. The study aimed to examine the feasibility of bile acids in distinguishing hepatocellular carcinoma from post hepatitis C virus-induced liver cirrhosis.

**Methods:** An ultra-performance liquid chromatography coupled with mass spectrometry measured 14 bile acids in patients with noncirrhotic post hepatitis C virus disease (n = 50), cirrhotic post hepatitis C virus disease (n = 50), hepatocellular carcinoma (n = 50), and control group (n = 50).

**Results:** The spectrum of liver disease was associated with a significant increase in many conjugated bile acids. The fold changes in many bile acid concentrations showed a linear trend with hepatocellular carcinoma > cirrhotic disease > noncirrhotic disease > healthy controls (*p* < 0.05). Receiver operating characteristic curve analysis revealed five conjugated acids TCA, GCA, GUDCA, TCDCA, GCDCA, that discriminated hepatocellular carcinoma from noncirrhotic liver patients (AUC = 0.85–0.96) with a weaker potential to distinguish it from chronic liver cirrhosis (AUC = 0.41–0.64).

**Conclusion:** Serum bile acids are associated primarily with liver cirrhosis with little value in predicting the progress of cirrhotic disease to hepatocellular carcinoma.

## Introduction

Several studies have associated liver cirrhosis with altered bile acid metabolism, and elevated serum bile acid concentrations can distinguish liver cirrhosis with higher sensitivity than the liver function tests (1–3). Bile acid synthesis and metabolism have a role in cellular processes related to carcinogenesis. Elevated intracellular concentrations of bile acids were associated with oxidative stress and DNA damage both in the adult and fetal liver (4,5). The disturbance in bile acid metabolism could be an early clue in the development of HCC, which is aggressive cancer, with around 90% of cases developing from pre-existing liver cirrhosis (6–8). Although the Child-Pugh classification is the cornerstone in prognostic evaluation of liver cirrhosis, however, in hepatocellular carcinoma (HCC) associated with cirrhosis, it may not provide direct evidence of the stage of the HCC, and bile acids might add as potential value as biomarkers for pathological progression in liver diseases (9). Early detection of HCC remains a challenge as HCC cases are usually diagnosed after the cancer has already progressed into advanced stages (5,10). Currently, no clinically approved alternatives to alpha-fetoprotein (AFP) that could form a noninvasive laboratory test for early detection of HCC. AFP demonstrates a low sensitivity as 40% of patients with HCC have normal AFP levels, and only 20% of patients with early HCC have elevated AFP levels (11). Des-gamma-carboxyprothrombin and lectin-bound AFP (AFP-L3), glypican-3, Osteopontin, or high c-met expression were hypothesized as alternative markers but, their sensitivity for HCC detection remains unsatisfactory especially, in small HCC lesions (12–15).

We used a systematic metabolomics approach applying ultra-performance liquid chromatography coupled with mass spectrometry (UPLC/MS) to hypothesis links between 14 bile acid profiles and malignant hepatic disease We tested this hypothesis in patients with a spectrum of liver disease, from healthy controls, to noncirrhotic liver disease, to HCV cirrhotic liver disease, and finally to HCC patients, expected to find a direct linear trend with significant differences between the four groups comprising the disease spectrum.

## Patients, Materials, and Methods

The study was conducted at the National Liver Institute hospital from October 2017 to August 2018 and complied with the ethical standards for human experimentation (Helsinki Declaration). The ethics committee of the National Liver Institute approved the protocol, and written consents were filled and signed by all the participants. The study enrolled three groups of patients and a control group. The noncirrhotic liver disease (NCLD) group (n = 50) enrolled patients with a documented previous HCV infection (positive anti-HCV antibody and PCR HCV- RNA) without any clinical or imaging (ultrasound and fibro scan) evidence of liver cirrhosis (16). The cirrhotic liver disease (CLD) group (n = 50) enrolled patients with liver cirrhosis secondary to previous HCV infection. The diagnosis of liver cirrhosis was established by ultrasound examination confirming the characteristic echogenic pattern of liver cirrhosis, fibro scan at ≥ 14.5 kPa, and positive anti-HCV antibody and HCV- RNA PCR tests. The HCC group (n = 50) enrolled patients with HCC complicating chronic HCV infection. The diagnosis of HCC was established by detecting focal hepatic lesion(s) on ultrasound examination, with elevated serum AFP >200 ng/ml and or detection of HCC on histological examination of the liver biopsy. CLD and HCC patients were further classified into Child-Pugh stages A, B and C (17). The control group (n = 50) enrolled normal, healthy subjects, matching the age and gender of the other groups with no clinical, laboratory, or imaging indication of liver cirrhosis or focal hepatic lesions. These subjects were also free from any other cancers, systemic disease as diabetes mellitus, obesity (BMI >30 kg/m^2^), or chronic cholecystitis, and they were abstinent from drug abuse and alcohol consumption.

Exclusion criteria include patients presenting with simultaneous HCV and hepatitis B virus (HBV) infection, chronic cholestasis, and extrahepatic obstructive gall bladder diseases. Patients presented with liver disease associated with severe renal or systemic diseases as cardiovascular, diabetes and obesity. No history of alcohol intake or illicit drug abuse in all patients enrolled in the study.

Blood samples were obtained from patients and control subjects after overnight fasting (8–12 h) by venipuncture technique, and the serum was stored at - 80 °C until analysis. An automatic biochemical analyzer (Beckman Ltd., London, United Kingdom) measured the blood chemistry as liver function tests, renal function tests, serum cholesterol, and triglycerides. Serum bile acids were prepared for UPLC/MS/MS as described (18,19) with modification. Briefly, 100 µl of the serum samples were treated with 400 µl of ice old 100% methanol to precipitate proteins. The mixture was centrifuged at 13,000 rpm for 15 min 50µl of the supernatant was mixed with 100 µl 0.001% formic acid, and 5 μl was injected into a C18 column (1.7 µm, 100 mm × 2.1 mm internal dimensions) of the ultra-performance liquid chromatography with the column temperature maintained at 50°C (Waters ACQUITY, Milford, MA). The individual bile acids were eluted by gradient at a flow rate of 0.5 ml/min, for 2 min with 80% mobile phase A (0.001 formic acid in water) and 20% mobile phase B (acetonitrile), then with a linear gradient of mobile phase B (20–30%) over 5 min followed by mobile phase B at (80%) for 8 min. The mass spectrometer had an electrospray source operated in the negative ion mode using the Multiple Reactions Monitoring (MRM). UPLC-MS raw data obtained with MRM mode were analyzed using Target Lynx application manager version 4.1 (Waters Corp., Milford, MA) to obtain the calibration equations and the quantitative concentration of each bile acid in the samples. Calibration curves and method assessment were processed by a freshly prepared 14 bile acids mixture standard solution was serially diluted to make seven standard calibration points ranging from (0.125 μmol/L to 20 μmol/L) other than the zero point. The quality control (QC) standards were also prepared to make low, mid, and high internal standard points (0.2, 2, and 20 μmol/L) in charcoal-stripped serum (19). Calibrators and QC standards, then, underwent the sample preparation process described before. Calibration curves showed that bile acids had a linear response, with a coefficient of determination (R2) ≥0.99. The recovery was evaluated by comparing the mean detector response of the extracted QC samples at 0.2, 2, and 20 μmol/L in four replicates to the mean detector response of the post-extracted serum blanks spiked at equal concentrations. The accuracy and precision were checked regularly using three replicates of freshly prepared QC standard samples at 0.2, 2, and 20 μmol/L concentration each day. Accuracy was calculated from the % Relative Error (RE) (% (measured-theoretical)/theoretical concentration). Precision was calculated from the relative standard deviation (%RSD = % standard deviation/mean). The developed UPLC-MS/MS assay method had the capability of quantitation of all the 14 bile acids included in the study. The assay performance was accurate and precise for bile acid analysis in the human serum (19).

Data were analyzed using IBM SPSS 23 (SPSS Inc., CA, United States). The nonparametric statistical Kruskal-Wallis test with the Mann-Whitney test was used to detect the significance in multiple comparisons. The linear contrast analysis was used to detect a direct trend across the different groups. Receiver operator characteristic (ROC) curves assessed the ability of serum bile acids to distinguish healthy subjects from patients with liver diseases. An area under the curve (AUC) ≥ 0.8 was considered a significant-good or excellent test to discriminate between two groups (20,21).

## Results

Table 1 summarizes the demographic, clinical, and laboratory parameters of the enrolled groups. Patients matched for age, gender, and body mass index (BMI) to control biological and lifestyle confounders. Neither age, gender, or BMI shows any significant differences across groups, all *p* > 0.05. All patients in the NCLD group had a well-compensated liver function. In the CLD group, 38 patients were Child-Pugh A, eight were stage B and four were stage C. In the HCC group, patients had either a single focal lesion (n = 19) or multiple focal lesions (n = 31), lymph node involvement (n = 7), distant metastasis (n = 4), but none had portal vein invasion. Of the HCC patients, 18 were Child-Pugh A, 17 were stage B and 15 were stage C. Unsurprisingly, all laboratory indices showed a significant linear trend with the disease spectrum, with numerous differences between groups. However, there was no statistically significant difference between the NCLD group and the control regarding direct bilirubin, total protein, haemoglobin, and the white cell count (all *p* > 0.05) whilst. The levels of direct bilirubin, GGT, ALP increased while albumin, total protein, haemoglobin, and platelets, decreased in cirrhotic patients compared to NCLD or NHC (all *p* < 0.05).

**TABLE 1:** Demographic and laboratory parameters of the enrolled groups.

	NHC N = 50	NCLD N = 50	CLD N = 50	HCC N = 50	LCA *p*-value
Age, mean (range)	45 (34–73)	46 (36–69)	46 (37–70)	46 (37–69)	0.05
BMI (kg/m^2^)	23 ± 2	24 ± 4	23 ± 2	25 ± 1	0.06
Sex					
Male	25 (50%)	19 (38%)	29 (58%)	15 (30%)	0.554^a^
Female	25 (50%)	31 (62%)	21 (42%)	35 (70%)	
AFP (µmol/l)	1.7 (1.3–2.1)	2.3 (1.9–3.2)	3.7 (2.3–6.7)^b^	68 (4.6–881.5)^b^	0.049
AST (IU/L)	21 ± 6	40 ± 34^b^	57 ± 35^b^	55 ± 29^b^	0.001
ALT (IU/L)	20 ± 9	4 ± 37^b^	42 ± 44^b^	40 ± 19^b^	0.004
Total bilirubin (µmol/l)	9 ± 4	11 ± 6^b^	26 ± 29^b^	25 ± 20^b^	0.001
Direct bilirubin (µmol/l)	4 ± 2.4	4 ± 3	21 ± 28^b^	14 ± 13^b^	0.001
Albumin (g/l)	40 ± 2	47 ± 3	35 ± 8^b^	33 ± 7^b^	0.001
Total protein (mg/l)	70 ± 9	80 ± 4	80 ± 7	70 ± 7^b^	0.001
GGT (IU/ml)	23 ± 13	35 ± 22^b^	71 ± 53^b^	73 ± 61^b^	0.001
ALP (IU/ml)	60 ± 23	75 ± 47	114 ± 57^b^	117 ± 49^b^	0.001
Haemoglobin (g/l)	134 ± 12	131 ± 15	119.8 ± 20^b^	120 ± 20^b^	0.001
Platelets × (10^9^/l)	291 ± 72	259 ± 91	132 ± 64^b^	129 ± 63^b^	0.001
WBCs × (10^9^/l)	7.3 ± 1	6.9 ± 1	5.4 ± 2^b^	5 ± 2^b^	0.001

NHC, normal healthy control; CLD, cirrhotic liver diseases; HCC, Hepatocellular carcinoma. LCA, linear contrast analysis. Data n (%); mean with SD; or median with IQR. BMI, body mass index; AST, aspartate transaminase; ALT, alanine transaminase; GGT, Gamma-Glutamyl transferase; ALP, alkaline phosphatase; WBCs, white blood cells.

a chi-squared test of trend.

b *p*-value < 0.05 indicates significance when NHC, compared to NCLD, CLD, and HCC.

The spectrum of liver disease was associated with significant increases in most of the bile acids (Table 2). Linear contrast analysis of the 14 bile acids across the different groups showed that all but TUDCA, GDCA, TLCA showed a statistically significant stepwise increase in the serum bile acids with the severity of the liver disease from NCLD to CLD to HCC, all *p* < 0.05. The fold change of bile acids relative to the control showed a significant pattern that HCC > CLD > NCLD and the increase in the fold was mainly prominent in conjugated bile acids. Eight bile acids (CA, CDCA, UDCA, TCA, GCA, GUDCA, TCDCA, and GCDCA) were significantly higher in CLD and HCC than in NHC or NCLD (all *p* < 0.05).

**TABLE 2:** Serum bile acids and fold changes across the different groups.

BA	NHC	NCLD	CLD	HCC	LCA, p	Fold relative to NHC		
						NCLD	CLD	HCC
TUDCA	0.04 ± 0.12	0.01 ± 0.02	0.09 ± 0.37	0.90 ± 5.17	0.26	0.3	2^a^	18^a^
TCA	0.01 ± 0.05	0.04 ± 0.09^a^	1.7 ± 2.7^a^	7.05 ± 16^a^	0.01	3^a^	113^a^	473^a^
GCA	0.24 ± 0.33	0.43 ± 0.77	3.77 ± 4.06^a^	8.4 ± 13^a^	0.01	2^a^	16^a^	35^a^
GUDCA	0.15 ± 0.2	0.07 ± 0.12^a^	5.2 ± 12.9^a^	9.4 ± 30^a^	0.01	0.5	35^a^	38^a^
TCDCA	0.11 ± 0.18	0.07 ± 0.15	4.77 ± 13.8^a^	9.1 ± 16^a^	0.01	0.7	44^a^	83^a^
TDCA	0.07 ± 0.15	0.02 ± 0.03	0.19 ± 0.36	1.6 ± 6 ^NS^	0.06	0.3	3^a^	24^a^
CA	0.20 ± 0.3	0.6 ± 1.7	0.5 ± 0.86^a^	1.7 ± 4^a^	0.01	3^a^	3^a^	9^a^
GCDCA	0.45 ± 0.66	0.86 ± 1.04^a^	7.7 ± 11.5^a^	12.5 ± 17^a^	0.01	2^a^	17^a^	29^a^
UDCA	0.04 ± 0.1	0.04 ± 0.08	1.56 ± 5.01^a^	1.99 ± 6^a^	0.01	1	38^a^	48^a^
GDCA	0.24 ± 0.36	0.26 ± 0.33	1.48 ± 2.9^a^	1.72 ± 7.2	0.07	1	6^a^	7^a^
TLCA	0.006 ± 0.028	0.001 ± 0.002^a^	0.02 ± 0.07^a^	0.08 ± 0.3^a^	0.07	0.3	4^a^	13^a^
CDCA	0.38 ± 0.51	0.73 ± 1.2	1.45 ± 1.91^a^	5.1 ± 14^a^	0.02	2^a^	4^a^	13^a^
DCA	0.15 ± 0.16	0.19 ± 0.11^a^	0.31 ± 0.40	0.25 ± 0.39	0.02	1	2^a^	2^a^
LCA	0.01 ± 0.03	0.03 ± 0.05^a^	0.05 ± 0.09^a^	0.11 ± 0.20^a^	0.01	3^a^	4^a^	9^a^

NHC, normal healthy control; NCLD, noncirrhotic liver disease; CLD, cirrhotic liver diseases; HCC, Hepatocellular carcinoma. LCA, linear contrast analysis. Values, mean ± standard deviation of bile acids (µM/L); F, Fold changes relative to NHC. TUDCA, tauroursodeoxycholic acid; TCA, taurocholic acid; GCA, glycholic acid; GUDCA, glycoursodeoxycholic acid; TCDCA, taurochenodeoxycholic acid; TDCA, taurodeoxycholic acid; CA, cholic acid; GCDCA, glycochenodeoxycholic acid; UDCA, ursodeoxycholic acid; GDCA, glycodeoxycholic acid; TLCA, taurolithocholic acid; CDCA, chenodeoxycholic acid; DCA, deoxycholic acid; and LCA, lithocholic acid.

a *p*-value < 0.05 indicates significance when NHC, compared to either NCLD, CLD, or HCC.

Table 3 summarizes the results of ROC curves of the 14 bile acids and their diagnostic performance. Conjugated bile acids TCA, GCA, GUDCA, TCDCA, and GCDCA showed the best diagnostic performance in separating HCC from NHC with AUCs, and in separating HCC from NCLD. Only GCA, TCDA and LCA discriminated HCC from CLD. However, although significant at *p* < 0.05, many failed to reach our pre-set level of practical significance, i.e. AUC ≥0.8, giving *p* < 0.01.

**TABLE 3:** Receiver operating characteristic curve for the 14 bile acids in the NHC, NCLD and HCC.

Bile acid	NHC vs. HCC		NCLD vs. HCC		CLD vs. HCC	
	AUC (95%CI)	*p*	AUC (95%CI)	*p*	AUC(95% CI)	*p*
TUDCA	0.61 (0.50–0.72)	0.06	0.67 (0.56–0.77)	<0.01	0.53 (0.41–0.65)	0.59
TCA	0.86 (0.78–0.94)	<0.01	0.80 (0.70–0.89)	<0.01	0.56 (0.44–0.67)	0.31
GCA	0.89 (0.82–0.96)	<0.01	0.86 (0.78–0.94)	<0.01	0.62 (0.51–0.73)	0.04
GUDCA	0.85 (0.77–0.93)	<0.01	0.89 (0.82–0.96)	<0.01	0.61 (0.50–0.72)	0.06
TCDCA	0.96 (0.92–1.00)	<0.01	0.97 (0.93–1.00)	<0.01	0.63 (0.52–0.74)	0.03
TDCA	0.52 (0.40–0.63)	0.78	0.56 (0.44–0.67)	0.33	0.49 (0.38–0.61)	0.87
CA	0.64 (0.53–0.75)	0.02	0.59 (0.47–0.70)	0.14	0.47 (0.36–0.59)	0.63
GCDCA	0.95 (0.90–1.00)	<0.01	0.91 (0.85–0.97)	<0.01	0.58 (0.46–0.69)	0.18
UDCA	0.64 (0.53–0.75)	0.01	0.59 (0.48–0.71)	0.10	0.45 (0.33–0.56)	0.36
GDCA	0.56 (0.44–0.68)	0.31	0.50 (0.38–0.63)	0.95	0.41 (0.30–0.53)	0.13
TLCA	0.65 (0.54–0.76)	0.01	0.53 (0.41–0.65)	0.63	0.50 (0.39–0.62)	0.98
CDCA	0.74 (0.65–0.84)	<0.01	0.69 (0.59–0.79)	<0.01	0.54 (0.42–0.65)	0.52
DCA	0.47 (0.35–0.59)	0.61	0.38 (0.26–0.50)	0.03	0.45 (0.33–0.56)	0.36
LCA	0.79 (0.70–0.88)	<0.01	0.61 (0.50–0.72)	0.05	0.63 (0.52–0.74)	0.02

NHC, normal healthy control; NCLD, non cirrhotic liver disease; CLD, cirrhotic liver diseases; HCC, Hepatocellular carcinoma. AUC, area under curve; CI, Confidence interval. TUDCA, tauroursodeoxycholic acid; TCA, taurocholic acid; GCA, glycholic acid; GUDCA, glycoursodeoxycholic acid; TCDCA, taurochenodeoxycholic acid; TDCA, taurodeoxycholic acid; CA, cholic acid; GCDCA, glycochenodeoxycholic acid; UDCA, ursodeoxycholic acid; GDCA, glycodeoxycholic acid; TLCA, taurolithocholic acid; CDCA, chenodeoxycholic acid; DCA, deoxycholic acid; and LCA, lithocholic acid.

Table 4 presents analysis of bile acids with the Child-Pugh grades in the HCC group by the nonparametric Kruskal-Wallis. Three bile acids GCA, TCDCA, and GCDCA, were linked to Child-Pugh class (*p* < 0.05), However further analysis, Mann-Whitney test revealed that GCA, TCDCA, and GCDCA in Class B were significantly higher than in either class A or C. A further caveat is that none of the bile acids showed a linear trend with the Child-Pugh stage, where in many cases levels were highest in those with intermediate disease i.e. stage B.

**TABLE 4:** Bile acid among different Child-Pugh classes of HCC.

Bile acid	CP	Median (IQR)	*p*-value	A vs. B	A vs. C	B vs. C
TUDCA	A	0.03 (0.001–0.13)	0.84	0.484	0.926	0.831
	B	0.07 (0.001–0.2)				
	C	0.01 (0.001–0.2)				
TCA	A	0.55 (0.001–2.1)	0.15	0.061	0.561	0.191
	B	5.80 (0.05–31)				
	C	0.70 (0.001–3)				
GCA	A	2.65 (0.9–7.6)	0.005	0.006	0.612	0.005
	B	11.2 (4.6–24)				
	C	2.50 (0.1–5.5)				
GUDCA	A	0.40 (0.28–2.4)	0.092	0.192	0.036	0.316
	B	0.80 (0.35–12.30				
	C	5.50 (0.3–13.6)				
TCDCA	A	0.86 (0.48–2.2)	0.001	0.001	0.043	0.064
	B	12.1 (2.1–33)				
	C	2.80 (0.7–9.2)				
TDCA	A	0.01 (0.001–0.1)	0.711	0.903	0.322	0.675
	B	0.01 (0.006–0.64)				
	C	0.03 (0.001–0.8)				
CA	A	0.13 (0.05–1.2)	0.729	0.778	0.444	0.591
	B	0.10 (0.08–3.75)				
	C	0.20 (0.1–0.72)				
GCDCA	A	3.3 (2.2–7.0)	0.001	0.001	0.759	0.004
	C	15.70 (5.7–31)				
	B	3.90 (0.9–10)				
UDCA	A	0.01 (0.001–0.36)	0.133	0.455	0.036	0.291
	B	0.10 (0.01–0.35)				
	C	0.13 (0.01–2.2)				
GDCA	A	0.46 (0.01–2.3)	0.315	0.16	0.316	0.51
	B	0.10 (0.01–1.1)				
	C	0.10 (0.1–1.3)				
TLCA	A	0.01 (0.001–0.05)	0.323	0.704	0.172	0.235
	B	0.01 (0.001–0.01)				
	C	0.01 (0.001–0.30)				
CDCA	A	0.65 (0.25–1.18)	0.289	0.133	0.527	0.325
	B	0.90 (0.4–6.5)				
	C	0.73 (0.22–4.1)				
DCA	A	0.10 (0.01–0.21)	0.371	0.448	0.41	0.185
	B	0.05 (0.01–0.17)				
	C	0.20 (0.01–0.80)				
LCA	A	0.02 (0.01–0.05)	0.133	0.105	0.096	0.391
	B	0.04 (0.01–0.10)				
	C	0.10 (0.01–0.20)				

NHC, normal healthy control; NCLD, noncirrhotic liver disease; CLD, cirrhotic liver diseases, HCC, Hepatocellular carcinoma. CP, Child-Pugh; CP A = 18, CP B = 17, CP C = 15, Values, Median and IQR, Interquartile Range (IQR) of bile acids (µM/L), Percentile 25 and 75%. Kruskal-Wallis test, *p*-value < 0.05 indicates significance comparison.

## Discussion

The study assessed the metabolic profile of 14 bile acids in patients with different stages of liver diseases from health, to NCLD, to CLD, and to HCC. The alterations in the serum bile acids level in the noncirrhotic patients compared to healthy controls were insignificant, suggesting that the liver is unaffected by relatively minor disease. Five conjugated bile acids TCA, GCA, GUDCA, TCDCA, and GCDCA, increased in cirrhotic patients compared to noncirrhotic disease. This reflect work by Yin Wan et al. who found GCA, GCDCA, TCA, and TCDCA significantly increased in cirrhotic patients, suggesting these bile acid could be biomarkers of progression liver cirrhosis (22).

In CLD and HCC, the level of conjugated bile acids was significantly higher than the unconjugated, suggesting that conjugated bile acids may reflect the progress of the chronic liver cirrhosis to HCC (23). An increase in the conjugated bile acids has long been observed in patients with hepatobiliary diseases such as viral hepatitis, cirrhosis, and cholangiocarcinoma (22), and the metabolic changes associated with the progression of liver cirrhosis to early stages of HCC include oxidative stress and abnormal metabolism of bile acids which trigger DNA damage and apoptosis (1,24,25). The process of bile acids conjugation results in less toxic and more water-soluble bile acid types, thus protecting against cellular damage from such toxic compound that triggers oxidative stress and stimulates cell death signaling (19). In the current study, there was a linear trend of increase in bile acids with the spectrum of the liver disease, reaching the highest level in HCC, yet bile acids did not distinguish HCC from CLD. Among the 14 bile acids, GCA, TCDCA, and GCDCA were significantly associated with the Child-Pugh grades but were higher in the B stage than A or C stages. Chen et al. reported that early-stage HCC was associated with elevated levels of conjugated bile acids, whereas levels of bile acids. were elevated to a lesser extent in patients with more advanced HCC (1). However, Wang et al. observed an increase in bile acids with the progress of liver cirrhosis. The reason for such changes is unknown, but it might be due to the disruption of liver function associated with tumorigenesis. A possible explanation for such discrepancies also might be related to intestinal microbial responsible for the metabolism of bile acids and the changes in enterohepatic circulation balance by the pathological progression of the HCV cirrhosis to HCC (9).

Several metabolomics studies have identified metabolite expression profile differences between HCC and healthy controls (1,26,27), but few have reported metabolomics profile differences between HCC and liver cirrhosis (28–31). Ressom et al. characterized the metabolic changes relating to HCC in patients with liver cirrhosis and found bile acids downregulated in HCC relative to cirrhosis (30). Xiao et al. showed a down-regulation of three bile acids, GCA, GDCA, and GCDCA, in HCC compared to liver cirrhosis (28). Chen et al. identified four bile acids CA, GCA, DCA, and GCDCA, which were altered differently in HCC with or without liver cirrhosis (1). A weakness with all these studies is that they have failed to address the full spectrum of liver disease, which present herein. Collectively, conjugated bile acids are associated with the progression of liver cirrhosis. High levels of conjugated bile acids in cirrhotic patients may be potential threat biomarkers for the occurrence of HCC in patients with early cirrhosis.

We acknowledge certain limitations. Patients were matched by demographic and clinical characteristics to control factors that may confound interpretation. However, other diseases such as diabetes, obesity, metabolic syndrome, cardiovascular disease, and gastrointestinal microbiota related to bile acids metabolism have not been considered, as their coexisting adds layers of complexity in the interpretation of the results of this metabolomics profiling (26,32–34). Therefore, further studies integrating HCC metabolomics data are needed to delineate the complicated relationship with the other diseases that might confound the results. Further limitations are that our data apply only to those whose liver disease is linked to hepatitis C virus infection, and that as our data is cross-sectional, we cannot be sure it genuinely reflects disease progression in an individual, although we speculate that this is indeed the case.

In conclusion, we characterized the metabolic profile of 14 bile acids in serum of patients with liver dysfunction ranging from non-cirrhosis, cirrhosis, and HCC using UPLC-MS/MS methods. The level of conjugated bile acids TCA, GCA, GUDCA, TCDCA, and GCDCA, were consistently higher in HCC than in NCLD and showed a tendency to be higher in HCC than CLD but without evident statistical significance. The work represents an advance in biomedical science because it shows that the increase in the serum bile acids level in patients with HCV-induced liver cirrhosis might serve as biomarkers for the progress of liver cirrhosis disease.

## Summary Table

### What is Known About This Subject

• Liver disease, such as cirrhosis, in characterised by altered bile acid metabolism.
• The role of serum bile acid in the diagnosis and prognosis of hepatocellular carcinoma (HCC) is controversial.

### What This Study Adds

• Many bile acids show a linear trend increase across the spectrum of liver disease, culminating in HCC.
• Conjugated bile acids TCA, GCA, GUDCA, TCDCA, and GCDCA discriminated HCC from NHC and NCLD, but not from CLD.
• Serum bile acids are associated primarily with liver cirrhosis but have little value in differentiating cirrhotic disease from HCC.

## Data Availability

The original contributions presented in the study are included in the article/supplementary material, further inquiries can be directed to the corresponding author.

## References

[1] Chen T Xie G Wang X Fan J Qiu Y Zheng X Serum and Urine Metabolite Profiling Reveals Potential Biomarkers of Human Hepatocellular Carcinoma. Mol Cel Proteomics (2011) 10(7)–004945. M110. 10.1074/mcp.M110.004945 PMC3134066 21518826

[2] Ciotti M Ciccozzi M Terrinoni A Jiang WC Wang CB Bernardini S. The COVID-19 Pandemic. Crit Rev Clin Lab Sci (2020) 57(6):365–88. 10.1080/10408363.2020.1783198 32645276

[3] Greco AV Mingrone G. Serum Bile Acid Concentrations in Mild Liver Cirrhosis. Clin Chim Acta (1993) 221(1-2):183–9. 10.1016/0009-8981(93)90032-y 8149635

[4] Baptissart M Vega A Maqdasy S Caira F Baron S Lobaccaro JM Bile Acids: from Digestion to Cancers. Biochimie (2012) 95(3):504–17. 10.1016/j.biochi.2012.06.022 22766017

[5] Davila JA Morgan RO Richardson PA Du XL McGlynn KA El-Serag HB. Use of Surveillance for Hepatocellular Carcinoma Among Patients with Cirrhosis in the United States. Hepatology (2010) 52(1):132–41. 10.1002/hep.23615 20578139 PMC3835698

[6] Moustafa T Fickert P Magnes C Guelly C Thueringer A Frank S Alterations in Lipid Metabolism Mediate Inflammation, Fibrosis, and Proliferation in a Mouse Model of Chronic Cholestatic Liver Injury. Gastroenterology (2012) 142(1):140–51. 10.1053/j.gastro.2011.09.051 22001865

[7] Chiang JYL Ferrell JM. Bile Acid Metabolism in Liver Pathobiology. Gene Expr (2018) 18(2):71–87. 10.3727/105221618x15156018385515 29325602 PMC5954621

[8] Barr DC Hussain HK. MR Imaging in Cirrhosis and Hepatocellular Carcinoma. Magn Reson Imaging Clin North America (2014) 22(3):315–35. 10.1016/j.mric.2014.04.006 25086932

[9] Wang X Xie G Zhao A Zheng X Huang F Wang Y Serum Bile Acids Are Associated with Pathological Progression of Hepatitis B-Induced Cirrhosis. J Proteome Res (2016) 15(4):1126–34. 10.1021/acs.jproteome.5b00217 25964117 PMC5660916

[10] El-Serag HB Alsarraj A Richardson P Davila JA Kramer JR Durfee J Hepatocellular Carcinoma Screening Practices in the Department of Veterans Affairs: Findings from a National Facility Survey. Dig Dis Sci (2013) 58(11):3117–26. 10.1007/s10620-013-2794-7 23868438 PMC3858077

[11] Zhao ZH Lai JKL Qiao L Fan JG. Role of Gut Microbial Metabolites in Nonalcoholic Fatty Liver Disease. J Dig Dis (2019) 20(4):181–8. 10.1111/1751-2980.12709 30706694

[12] Khalil A Elgedawy J Faramawi MF Elfert A Salama I Abbass A Plasma Osteopontin Level as a Diagnostic Marker of Hepatocellular Carcinoma in Patients with Radiological Evidence of Focal Hepatic Lesions. Tumori (2013) 99(1):100–7. 10.1700/1248.13796 23549008

[13] Zhu N Zhang D Wang W Li X Yang B Song J A Novel Coronavirus from Patients with Pneumonia in China, 2019. N Engl J Med (2020) 382(8):727–33. 10.1056/NEJMoa2001017 31978945 PMC7092803

[14] Taura N Ichikawa T Miyaaki H Ozawa E Tsutsumi T Tsuruta S Frequency of Elevated Biomarkers in Patients with Cryptogenic Hepatocellular Carcinoma. Med Sci Monit (2013) 19:742–50. 10.12659/MSM.889361 24008520 PMC3775616

[15] Laflaquière B Leclercq G Choey C Chen J Peres S Ito C Identifying Biomarkers of Wharton's Jelly Mesenchymal Stromal Cells Using a Dynamic Metabolic Model: The Cell Passage Effect. Metabolites (2018) 8(1):18. 10.3390/metabo8010018 PMC5876007 29495309

[16] Schütte K Schulz C Poranzke J Antweiler K Bornschein J Bretschneider T Characterization and Prognosis of Patients with Hepatocellular Carcinoma (HCC) in the Non-cirrhotic Liver. BMC Gastroenterol (2014) 14:117. 10.1186/1471-230x-14-117 24990270 PMC4098694

[17] Pugh RN Murray-Lyon IM Dawson JL Pietroni MC Williams R. Transection of the Oesophagus for Bleeding Oesophageal Varices. Br J Surg (1973) 60(8):646–9. 10.1002/bjs.1800600817 4541913

[18] Kimhofer T Fye H Taylor-Robinson S Thursz M Holmes E. Proteomic and Metabonomic Biomarkers for Hepatocellular Carcinoma: a Comprehensive Review. Br J Cancer (2015) 112(7):1141–56. 10.1038/bjc.2015.38 25826224 PMC4385954

[19] Luo L Aubrecht J Li D Warner RL Johnson KJ Kenny J Assessment of Serum Bile Acid Profiles as Biomarkers of Liver Injury and Liver Disease in Humans. PLoS One (2018) 13(3):e0193824. 10.1371/journal.pone.0193824 29513725 PMC5841799

[20] Akobeng AK. Understanding Diagnostic Tests 3: Receiver Operating Characteristic Curves. Acta Paediatr (2007) 96(5):644–7. 10.1111/j.1651-2227.2006.00178.x 17376185

[21] Fischer JE Bachmann LM Jaeschke R. A Readers' Guide to the Interpretation of Diagnostic Test Properties: Clinical Example of Sepsis. Intensive Care Med (2003) 29(7):1043–51. 10.1007/s00134-003-1761-8 12734652

[22] Neale G Lewis B Weaver V Panveliwalla D. Serum Bile Acids in Liver Disease. Gut (1971) 12(2):145–52. 10.1136/gut.12.2.145 5548561 PMC1411536

[23] Yang J Zhao X Liu X Wang C Gao P Wang J High Performance Liquid Chromatography−Mass Spectrometry for Metabonomics: Potential Biomarkers for Acute Deterioration of Liver Function in Chronic Hepatitis B. J Proteome Res (2006) 5(3):554–61. 10.1021/pr050364w 16512670

[24] Gowda GN. Human Bile as a Rich Source of Biomarkers for Hepatopancreatobiliary Cancers. Biomarkers Med (2010) 4(2):299–314. 10.2217/bmm.10.6 20406071

[25] Agosti P Sabbà C Mazzocca A. Emerging Metabolic Risk Factors in Hepatocellular Carcinoma and Their Influence on the Liver Microenvironment. Biochim Biophys Acta (Bba) - Mol Basis Dis (2018) 1864(2):607–17. 10.1016/j.bbadis.2017.11.026 29197664

[26] Patterson AD Maurhofer O Beyoğlu D Lanz C Krausz KW Pabst T Aberrant Lipid Metabolism in Hepatocellular Carcinoma Revealed by Plasma Metabolomics and Lipid Profiling. Cancer Res (2011) 71(21):6590–600. 10.1158/0008-5472.can-11-0885 21900402 PMC3206149

[27] Liu N Feng J Lv Y Liu Q Deng J Xia Y Role of Bile Acids in the Diagnosis and Progression of Liver Cirrhosis: A Prospective Observational Study. Exp Ther Med (2019) 18(5):4058–66. 10.3892/etm.2019.8011 31611941 PMC6781791

[28] Xiao JF Varghese RS Zhou B Nezami Ranjbar MR Zhao Y Tsai TH LC-MS Based Serum Metabolomics for Identification of Hepatocellular Carcinoma Biomarkers in Egyptian Cohort. J Proteome Res (2012) 11(12):5914–23. 10.1021/pr300673x 23078175 PMC3719870

[29] Wang B Chen D Chen Y Hu Z Cao M Xie Q Metabonomic Profiles Discriminate Hepatocellular Carcinoma from Liver Cirrhosis by Ultraperformance Liquid Chromatography-Mass Spectrometry. J Proteome Res (2012) 11(2):1217–27. 10.1021/pr2009252 22200553

[30] Ressom HW Xiao JF Tuli L Varghese RS Zhou B Tsai TH Utilization of Metabolomics to Identify Serum Biomarkers for Hepatocellular Carcinoma in Patients with Liver Cirrhosis. Anal Chim Acta (2012) 743:90–100. 10.1016/j.aca.2012.07.013 22882828 PMC3419576

[31] Zhou L Ding L Yin P Lu X Wang X Niu J Serum Metabolic Profiling Study of Hepatocellular Carcinoma Infected with Hepatitis B or Hepatitis C Virus by Using Liquid Chromatography-Mass Spectrometry. J Proteome Res (2012) 11(11):5433–42. 10.1021/pr300683a 22946841

[32] Hrncir T Hrncirova L Kverka M Tlaskalova-Hogenova H. The Role of Gut Microbiota in Intestinal and Liver Diseases. Lab Anim (2019) 53(3):271–80. 10.1177/0023677218818605 30580671

[33] Tang WW Hazen SL. Dietary Metabolism, Gut Microbiota and Acute Heart Failure. Heart (2016) 102(11):813–4. 10.1136/heartjnl-2016-309268 26980719 PMC5659351

[34] Kolodziejczyk AA Zheng D Shibolet O Elinav E. The Role of the Microbiome in NAFLD and NASH. EMBO Mol Med (2019) 11(2). 10.15252/emmm.201809302 PMC6365925 30591521

